# Characterisation of Pellicles Formed by *Acinetobacter baumannii* at the Air-Liquid Interface

**DOI:** 10.1371/journal.pone.0111660

**Published:** 2014-10-31

**Authors:** Yassine Nait Chabane, Sara Marti, Christophe Rihouey, Stéphane Alexandre, Julie Hardouin, Olivier Lesouhaitier, Jordi Vila, Jeffrey B. Kaplan, Thierry Jouenne, Emmanuelle Dé

**Affiliations:** 1 Unité Mixte de Recherche 6270 CNRS - Laboratory “Polymères, Biopolymères, Surfaces”, University of Rouen, Mont-Saint-Aignan, France; 2 Laboratory of “Microbiologie Signaux et Micro-Environnement” - Equipe d’Accueil 4312, University of Rouen, Evreux, France; 3 Department of Microbiology, Hospital Clinic, Barcelona, Spain; 4 Department of Biology, American University, Washington, District of Columbia, United States of America; Loyola University Medical Center, United States of America

## Abstract

The clinical importance of *Acinetobacter baumannii* is partly due to its natural ability to survive in the hospital environment. This persistence may be explained by its capacity to form biofilms and, interestingly, *A. baumannii* can form pellicles at the air-liquid interface more readily than other less pathogenic *Acinetobacter* species. Pellicles from twenty-six strains were morphologically classified into three groups: I) egg-shaped (27%); II) ball-shaped (50%); and III) irregular pellicles (23%). One strain representative of each group was further analysed by Brewster’s Angle Microscopy to follow pellicle development, demonstrating that their formation did not require anchoring to a solid surface. Total carbohydrate analysis of the matrix showed three main components: Glucose, GlcNAc and Kdo. Dispersin B, an enzyme that hydrolyzes poly-*N*-acetylglucosamine (PNAG) polysaccharide, inhibited *A. baumannii* pellicle formation, suggesting that this exopolysaccharide contributes to pellicle formation. Also associated with the pellicle matrix were three subunits of pili assembled by chaperon-usher systems: the major CsuA/B, A1S_1510 (presented 45% of identity with the main pilin F17-A from enterotoxigenic *Escherichia coli* pili) and A1S_2091. The presence of both PNAG polysaccharide and pili systems in matrix of pellicles might contribute to the virulence of this emerging pathogen.

## Introduction

A biofilm is an organized community of bacterial cells surrounded by a protective self-secreted matrix of extracellular polymeric substances (EPS) [1], [2]. Biofilms attached to biotic or abiotic surfaces have been extensively studied. Nevertheless, bacterial aggregation can also take place at the air-liquid interface and in suspensions [3]. The biofilm formed at the air-liquid interface, generally referred to as “pellicle”, is a floating structure that requires a high organization due to the lack of a solid surface for initial attachment [4], [5].

An important component of the biofilm is the EPS matrix, a protective cover that maintains a cohesive structure and interacts with the external environment to allow the entrance of specific substances. It can act as a recycling centre to keep lysed cells and nutrients available for the bacterial community [6], [7]. The EPS matrix is mainly composed of polysaccharides, proteins, nucleic acids and lipids [6], [7]. Some of these molecules such as cell motility-associated appendages, fimbriae or pili, contribute to the initial stages of biofilm formation [7], [8]. The matrix is highly hydrated preventing biofilm desiccation and it may also contribute to antimicrobial resistance by decreasing the transport of these substances into the biofilm [1]. These characteristics are very important especially in nosocomial pathogens such as *Acinetobacter baumannii* because the biofilm gives them a protection from the hospital environment.

Over the last two decades, *A. baumannii* has emerged as a problematic opportunistic pathogen associated with nosocomial infections, such as pneumonia, bacteraemia or meningitis [9]–[12]. This species has been considered the paradigm of multiresistant bacteria due to its remarkable capacity to acquire mechanisms of resistance to antimicrobial agents. Moreover, its ability to persist in the hospital environment accounts for its emergence. This persistence and resistance to desiccation could be directly associated to biofilm formation [13], [14]. Indeed, *A. baumannii* can attach to biotic and abiotic surfaces in a process that has been associated with the presence of several factors: the pili assembly systems, the production of the Bap (Biofilm associated protein) surface-adhesion protein and the autotransporter Ata [15]–[17]. OmpA, the major outer membrane protein is also required for attachment to epithelial cells [18] and type IV fimbriae promote bacterial motility, enhancing bacterial adhesion [19]. Although most attention has been focused on the biofilm formed on solid surfaces, *A. baumannii* also forms thick pellicles at the air-liquid interface [20]–[22], a favourable niche because bacteria can obtain nutrients from the liquid media and oxygen from the air [5]. Note that this type of biofilm has been mostly associated to the more pathogenic *Acinetobacter* spp. [21] and as such its characterisation, especially the EPS matrix, is important to understand the interactions between the pellicle and the external environment.

This study aimed to explore and characterize *A. baumannii* pellicles and their EPS matrix. These structures were morphologically examined using different microscopy approaches and clustered into three different groups. A representative sample from each morphological group was studied in depth to determine the principal components of the EPS matrix *i.e.* the polysaccharide and proteins secreted to form this protective cover.

## Materials and Methods

### Bacterial strains & growth conditions

Eighty-six epidemiologically unrelated *A. baumannii* clinical isolates (Table S1) were screened in this study: 81 isolates collected in Spain during the GEIH-Ab2000 project [23], [24]; 2 isolates from the ICU in Hospital Charles Nicolle (Rouen, France); 3 isolates from the Hospital Clinic (Barcelona, Spain).

Pellicles were grown at 25°C in Mueller Hinton Broth (MHB) (Oxoid, France) or in T-broth medium (10 g/L bacto tryptone, 5 g/L NaCl) supplemented with 20 µg/ml congo red (CR-TB) to examine the production of cellulose [25] using initial inocula equivalent to an OD_600_ value of 0.01.

### Pellicle formation assay

**Figure 1:**
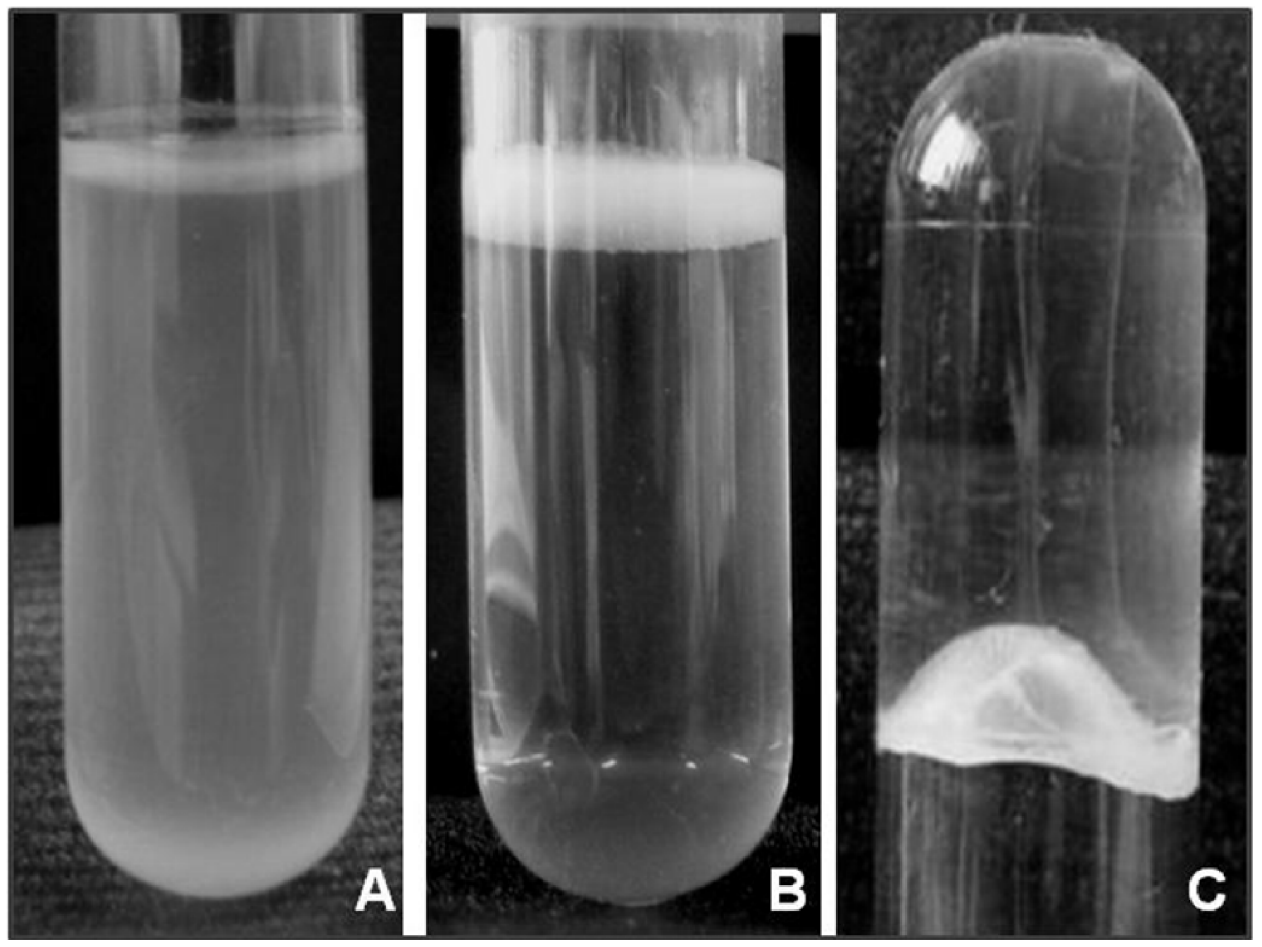
Biofilm formation by *A. baumannii*. **A)** Strain negative for pellicle formation (turbid culture); **B)** Strain forming a pellicle on the top of the liquid media (culture broth transparent); **C)** Inverted tube to examine pellicle strength.

Standing 2 mL cultures in MHB were grown for 72 h in polystyrene tubes (Ø 13 mm × H 75 mm). Pellicle formation was identified visually (Figure 1); isolates were considered positive when the surface of the culture was covered with an opaque layer. Pellicle cohesion was examined by manually inverting the tubes (Figure 1C). The experiments were performed in duplicate at three independent time-points.

### Microscopy analyses

#### Inversed Optical Microscopy

Standing 2 mL cultures in CR-TB were grown for 24 h in 24-well polystyrene plates and examined using a LEICA DM LM (Wetzlar, Germany) at ×5 amplification in the inverse mode. Photographs were taken with a Sony CMA-D2 camera, (Zaventem, Belgium) and the software Archimed (Microvision Instruments, Evry, France).

#### Brewster Angle Microscopy (BAM)

Brewster angle microscopy is an optical microscopy technique used to visualize materials located at an interface without the use of optical probes. It is based on the effect that no reflection occurs when a clean surface is lighted with p-polarized incoming light at the Brewster angle. The presence of materials at the interface leads to a partial light reflection allowing their visualization. Standing 15 mL cultures in MHB were grown for 24 h in 6-well polystyrene plates. The microscope used was a BAM 2 plus (NFT, Germany) with 2 µm resolution. Images were taken at different growth times to monitor pellicle evolution and are presented after a deinterlacing process to remove artefacts. Bacterial clusters were counted manually by examining the digital images superimposed by a micrometer grid. This allowed us to evaluate the number and size of the bacterial clusters.

#### Scanning Electron Microscopy (SEM)

Standing 7 mL cultures in MHB were grown for 24 h in 6-well polystyrene plates. To visualize the liquid-facing interface, pellicles formed on the surface of the liquid medium were recovered with collodion-coated cover slides as previously described by Henk *et al*. [26]. To visualize the biofilm air side, a non-coated cover slide was introduced, allowing the pellicle to sit on it. Samples were examined by SEM as previously described by Collet *et al.* [27].

#### Atomic Force Microscopy (AFM)

Water and air-facing sides of 24-h grown pellicles were visualized by AFM as described by Marti *et al.* [28]. AFM imaging was performed by using a Nanoscope III Multimode microscope (Veeco instrument, Santa Barbara, Ca, USA) with a 100 µm piezoelectric scanner. Imaging was achieved in the air in the contact mode. The cantilevers used were characterized by a low spring constant of about 0.06 N/m and were equipped with sharpened Si-tips. All the measurements were performed with the feedback loop on (constant force from 10^−9^ to 10^−8^ N). All images are presented in the height mode (black and white palette for height: dark for low zones, light for high zones) and are top-view images. A flatten operation was usually done on all images. This was achieved either with the nanoscope software (flatten order 2 or 3) or using the free Gwiddion AFM software downloadable at http://gwyddion.net/ (three points levelling, polynomial background order 2).

### Bacterial hydrophobicity

Bacterial adherence to hexadecane was adapted from Rosenberg *et al.* [29]. After an overnight planktonic culture (10 mL) at 25°C, bacteria were harvested by centrifugation (1500×g for 15 min) and the resulting pellet was washed and resuspended in sterile phosphate buffered saline solution (PBS) to a final absorbance value, A_400_ between 1.5 and 1.6. To 1.2 mL of bacterial suspension, 0.2 mL of Hexadecane (Acros Organics, France) were added and vortexed vigorously for 2 min. After 15 min standing at room temperature to allow phase separation, the absorbance was measured again. Bacterial hydrophobicity (%) was calculated as follows:
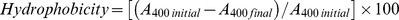



### Pellicle and crude matrix isolation

Biofilm matrix isolation and purification were adapted from Friedman *et al.* [30] with some modifications. Standing 1 L bacterial cultures on MHB in 2 L glass Erlenmeyers were inoculated to an initial OD_600_ value of 0.01 and left at 25°C without shaking for 5 days. Pellicles were recovered from the top of the culture with a 10 mL pipette, avoiding the culture medium, and washed once with dH_2_O. Pellets were resuspended with 10 mL of 1M NaOH, vortexed 30 s every 2 min for 15 min and centrifuged in a Beckman ultracentrifuge (Optima L-90 K Ultracentrifuge, CA, USA) at 19,000*×g* (70 Ti Rotor) for 1 h at 4°C. Supernatants were filtered through a 0.45 µm cellulose filter and neutralized with concentrated HCl. The matrix was precipitated at 4°C overnight, after addition of 3 volumes of cold 95% ethanol, centrifuged to recover the precipitated matrix, washed with 70% ethanol and then dried. Purified matrix was resuspended in dH_2_O and dialyzed overnight against water.

### Protein and polysaccharide quantification

Protein concentrations were determined using either the DC-protein assay (Bio-Rad, France) or the micro-BCA method from Pierce (Fisher, France). Quantification of neutral sugars was estimated by the modified phenol-sulfuric acid method [31] using Glucose (Glc), Galactose (Gal) and 3-Deoxy-manno-oct-2-ulopyranosonic acid (Kdo) standard curves. Briefly, 30 µL of 5% phenol aqueous solution (w/v) were automatically added (PerkinElmer: JANUS automated workstation, Courtaboeuf, France) to 30 µL of diluted purified pellicles (in triplicate) or standards solutions in a quartz 96-well microplate (Hellma, 730.009-QG). The plate was stirred for 1 min at 500 rpm on shaker (IKA, TTS 3 shaker with microtiter attachment) placed in controlled mechanical ventilation area. Then, 180 µL concentrated sulphuric acid (95%) were added and the plate was incubated in the oven for 20 minutes at 110°C. Finally, the plate was stirred for 5 min at 200 rpm and cooled to room temperature before reading the absorbance at 490 nm (A_490_) (Victor 3 spectrophotometer, PerkinElmer, Courtaboeuf, France). According to the monosaccharide composition of the EPS matrices and the linear responses of the Dubois assay for the standard sugars solutions, the quantity of detectable unmodified sugars (Q in mg) in the tested solution was calculated according to the following equation:
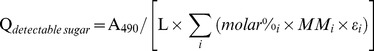
where *i* is one detectable sugar and *molar*%*_i_*, *MM_i_* and *ε_i_* are respectively, the molar percentage of sugar as determined by GC experiment, the molar mass and the extinction coefficient of the phenol-sugar condensed obtained from standard sugars calibration curves. L is constant in the reading process and the expression can be simplified to:



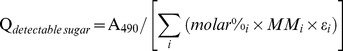



### Monosaccharide composition

25 µL of 4 mM inositol solution were added as internal standard to 500 µL of purified pellicles. Samples were freeze-dried and submitted to 16 h methanolysis at 80°C with 200 µL of 1 M methanolic-HCl. After evaporation of methanol, samples were re-acetylated by addition of 20 µL anhydrous acetic anhydride and 20 µL pyridine. The resulting N-acetyl methyl glycosides (methyl ester) were dried, converted into their trimethylsilyl derivatives and separated by gas chromatography (GC). The gas chromatograph (Varian GC3800, Les Ullis, France) was equipped with a flame ionization detector, a WCOT fused silica capillary column as stationary phase (Varian CP-Sil 5 CB length 25 m, i.d. 0.25 mm, film thickness 0.25 µm) and helium as gas vector (constant pressure 20 psi). The oven temperature program was: 2 min at 120°C, 10°C/min to 160°C, and 1.5°C/min to 220°C and then 20°C/min to 280°C. Sugar quantification was done by integration of peaks and determination of the corresponding molar values using response factors established with standard monosaccharides. Quantity of each carbohydrate is expressed in molar percentage from derivatization of 1 mg of total sugars and results from the analysis of three pellicles from each strain and a technical duplicate.

### Dispersin B effect on pellicle formation

Pellicles from each morphogroup (*i.e*., A061, A077 and A132 strains) were grown on 24-wells plates in MHB with or without 50 µg/mL Dispersin B for 24 h at 25°C without shaking. Pellicle formation was quantified according to the protocol described by O’Toole and Kolter [32]. For testing the activity of Dispersin B on 24-h pellicles, 50 µg/mL of the enzyme were added and pellicle formation was similarly quantified after 24 h of additional growth. All experiments were performed at least in triplicate. Statistical analyses were performed using Prism Graph Pad 4 software and significant differences between pellicles growth with or without Dispersin B were assessed by *P* values.

### Secreted protein identification

Purified matrixes were treated with 0.02% cellulase (Sigma-Aldrich, Lyon, France) at 37°C overnight with shaking. Samples were separated by discontinuous SDS-polyacrylamide gel electrophoresis (4.5% stacking, 15% separating gel) and gels were stained with 0.1% (wt/vol) Coomassie brilliant blue G 250 solution. Excised bands were washed several times with water and dried for 2 hours. Trypsin digestion was performed as described by Marti *et al*. [28]. Identification of secreted proteins was performed by LC-MS/MS. All experiments were done on a LTQ-Orbitrap Elite (Thermo Scientific) coupled with an Easy nano-Liquid Chromatography II system (Thermo Scientific). Tryptic peptides were eluted from 2 to 55% of B solution (0.1% formic acid in acetonitrile) over 14 min, from 55 to 100% of B solution in 1 min and 10 min at 100% of B solution. The samples were analysed using the CID (Top20) method. Raw data files were processed using Proteome Discoverer 1.3 software (Thermo Scientific). Peak lists were searched using the MASCOT search engine (Matrix Science) against the database *A. baumannii* ATCC17978 protein sequences at http://www.ncbi.nlm.nih.gov. Database searches were performed with the following parameters: 2 missed trypsin cleavage sites allowed; variable modifications: carbamidomethylation of cystein, oxidation of methionine. Quantitation was based on label-free approach using spectral counting (peptide spectral match – PSM). Protein relative abundance was expressed after minimum-maximum normalization method.

## Results

### Pellicle formation

Culture medium (2 mL) was inoculated with a low bacterial cell density (OD_600_ = 0.01) and grown at 25°C without shaking. After 24 hours, a thin pellicle started to form at the surface of the liquid media; this pellicle grew in thickness and by the end of the third day, an opaque and solid structure covered the whole liquid surface. Eighty-six *A. baumannii* clinical isolates were visually analysed for pellicle formation and twenty-six (30%) pellicle-forming isolates were separated for further analysis (Table S1). Pellicles were diverse depending on the isolates, with no growth difference between MHB or CR-TB, and were characterized as thick pellicles at the air-liquid interface attached to the wall of the tube, with transparent liquid media underneath, in contrast to negative samples that grew as turbid cultures with no pellicle attached to the tube (Figure 1A&B). Some pellicles were so strong that they could support, for several minutes, the weight of the liquid medium (2 g) when the tube was inverted (Figure 1C). Bacterial growth on congo-red containing agar plates was not useful to differentiate between pellicle-forming and non-forming strains (data not shown).

### Pellicle morphology

**Figure 2:**
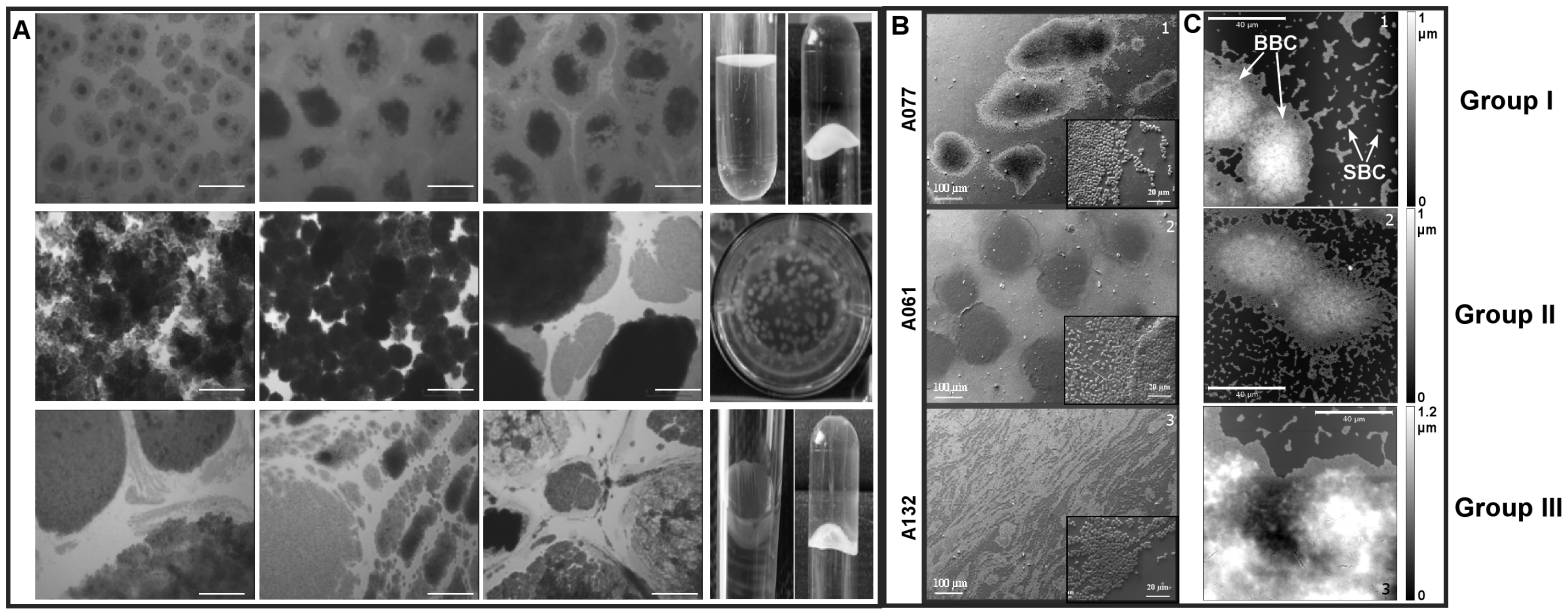
Different morphogroups of pellicles formed by *A. baumannii:* Group I (egg-shaped); Group II (ball-shaped); Group III (irregular pellicles). Section **A**: Pellicles were grown for 24 h in CR-TB and examined by inverse optical microscopy (three examples of strains from each morphogroup). White scale bar = 200 µm. Pellicles formed by strains from Group I and Group III were strong enough to support the weight of the medium; Section **B**: SEM, insets with enlarged scale; Section **C**: AFM; 100 µm×100 µm images taken after 48 h (A132) and 24 h (A061; A077) growth to be in a similar development stages of pellicles. Big bacterial clusters (BBC) and small bacterial clusters (SBC) are shown by arrows.

Twenty-six non related pellicle-forming *A. baumannii* isolates were classified by inverse optical microscopy into three different morphologic groups named from their appearance (Figure 2A, Table S1): ‘egg-shaped’ for its similarity with fried eggs observed by inverse microscopy, ‘ball-shaped’ for the presence of ball-like aggregations in liquid media, and ‘irregular’ pellicles for the differences in size and shape of the bacterial aggregates. In the ball-shaped group, non-associated bacterial aggregates can be observed floating on the surface of the liquid medium. These balls remained attached to a thin bacterial layer that covered the whole surface. It could be observed after removing the liquid medium underneath. The ball-shaped morphology was the most common, with 50% of the strains; the remaining strains were equally divided into the other two groups (7 and 6 strains, respectively).

A representative strain from each morphologic group was selected for further analyses by SEM and AFM (Figure 2B&C). SEM images of A077 (Group I: egg-shaped); A061 (Group II: ball-shaped); A132 (Group III: irregular pellicle) confirmed the morphologies already observed. AFM images allowed to assess the topography of the different morphotypes and showed big bacterial clusters surrounded by smaller ones (<50 bacteria) with a single layer in height (Figure 2C). A 3D comparison showed that A061 formed the most homogeneous pellicle with fairly round and uniform hills, A077 pellicle was less uniform and A132 pellicle was completely heterogeneous in height. For all the strains, the big bacterial clusters were surrounded by a layer with a height corresponding to a single bacterium. The width of this layer reached 4 to 9 µm for A061 and A077, when it could spread up to 20 µm for A132.

### Pellicle growth by BAM

**Figure 3:**
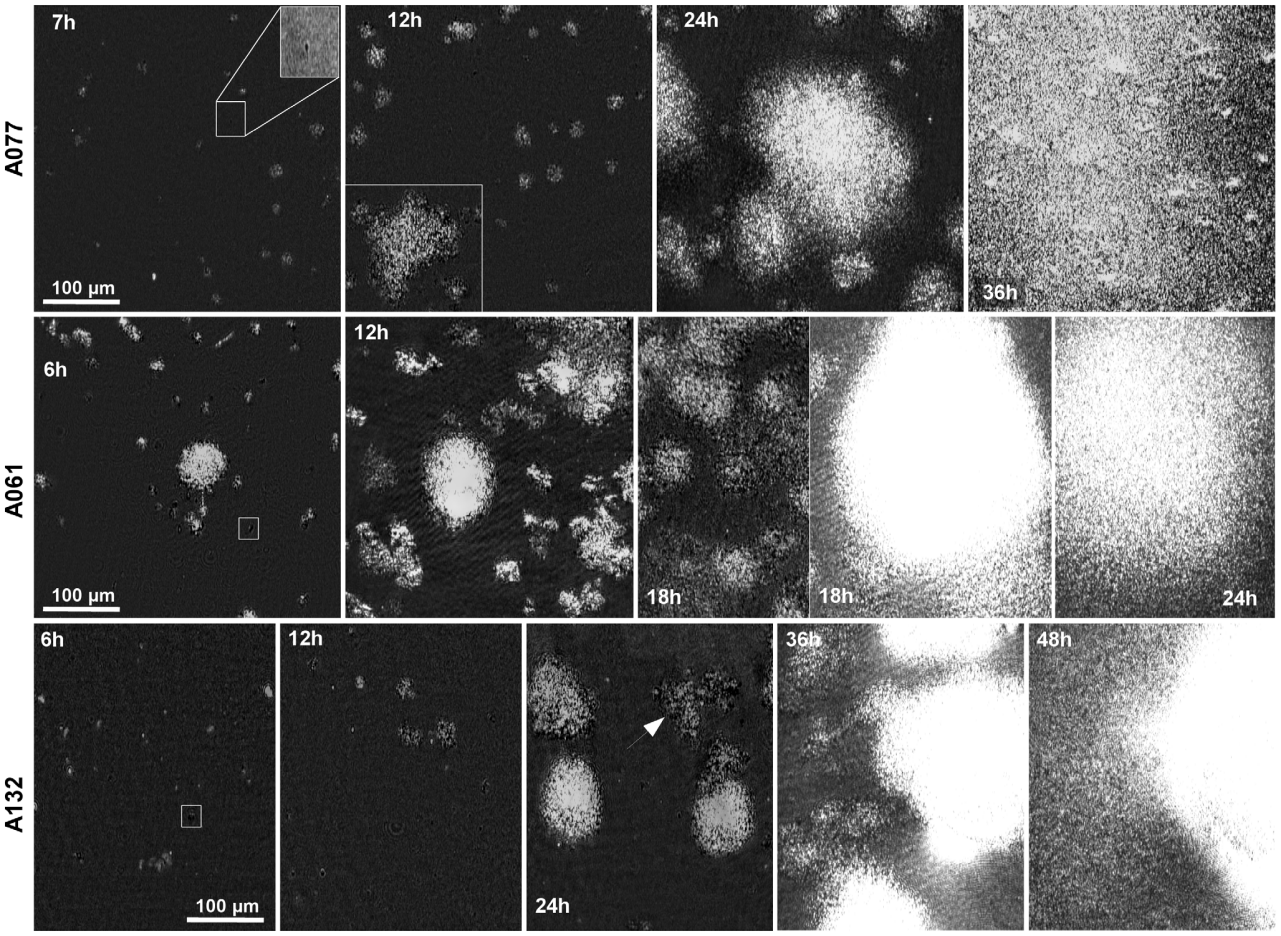
Brewster Angle Microscopy to follow *A. baumannii* pellicle development. Squared boxes showed initial dark clusters (for A077, the insert box is obtained with a two-fold magnification and an enhanced contrast). White arrow points large dark one-bacteria-in-height clusters.

Images taken few minutes after placing the bacterial suspension in the plate showed that the light was slightly reflected because of the organic monolayer (lipids and amphiphile proteins from the growth medium) already formed at the air-liquid interface. This monolayer remained stable and static over the first hour. After two hours, bright and dark objects started to appear on the surface. Actually, bacteria clusters organized in the plane of the surface lead locally to a decrease in light reflection and then correspond to the dark objects. When bacteria start to assemble as three dimensional clusters, the incoming laser light is locally no longer at the Brewster angle leading to a high light reflection. This is responsible for the observed bright objects. The area of these initial clusters was around 25 µm^2^ (20 to 30 pixels). Then, these clusters were composed of 20 to 30 bacteria. Bright and dark clusters grew in size during the next hours with a widespread conversion from dark into bright (Figure 3, see squared boxes).

#### Isolate A061 (Group II)

For 6 h, cluster growth (size and reflective intensity) was continuous, growing as independent units and also merging with nearby clusters. The total number of clusters was stable for at least the next 6 hours, with new clusters still formed at this stage of pellicle formation (see Figure 3, A061- 6 h & 12 h). The overall surface was covered by the pellicle after 36 h.

#### Isolates A077 (Group I) and A132 (Group III)

Pellicle formation was more heterogeneous up to the 18 h of growth for A077 and 36 h for A132 (Figure 3, A077- 12 h & A132- 24 h) and clusters were characterized by variable area and height. This phenomenon was even more evident for the A132 strain, which at 24 h growth, still presented large dark one bacterium in height clusters (see arrow in Figure 3, A132- 24 h) as well as large bright clusters, showing a certain level of competition between the building of the biofilm in the plane and in height. This could be responsible for the slower rate of pellicle formation.

### Bacterial hydrophobicity

**Figure 4:**
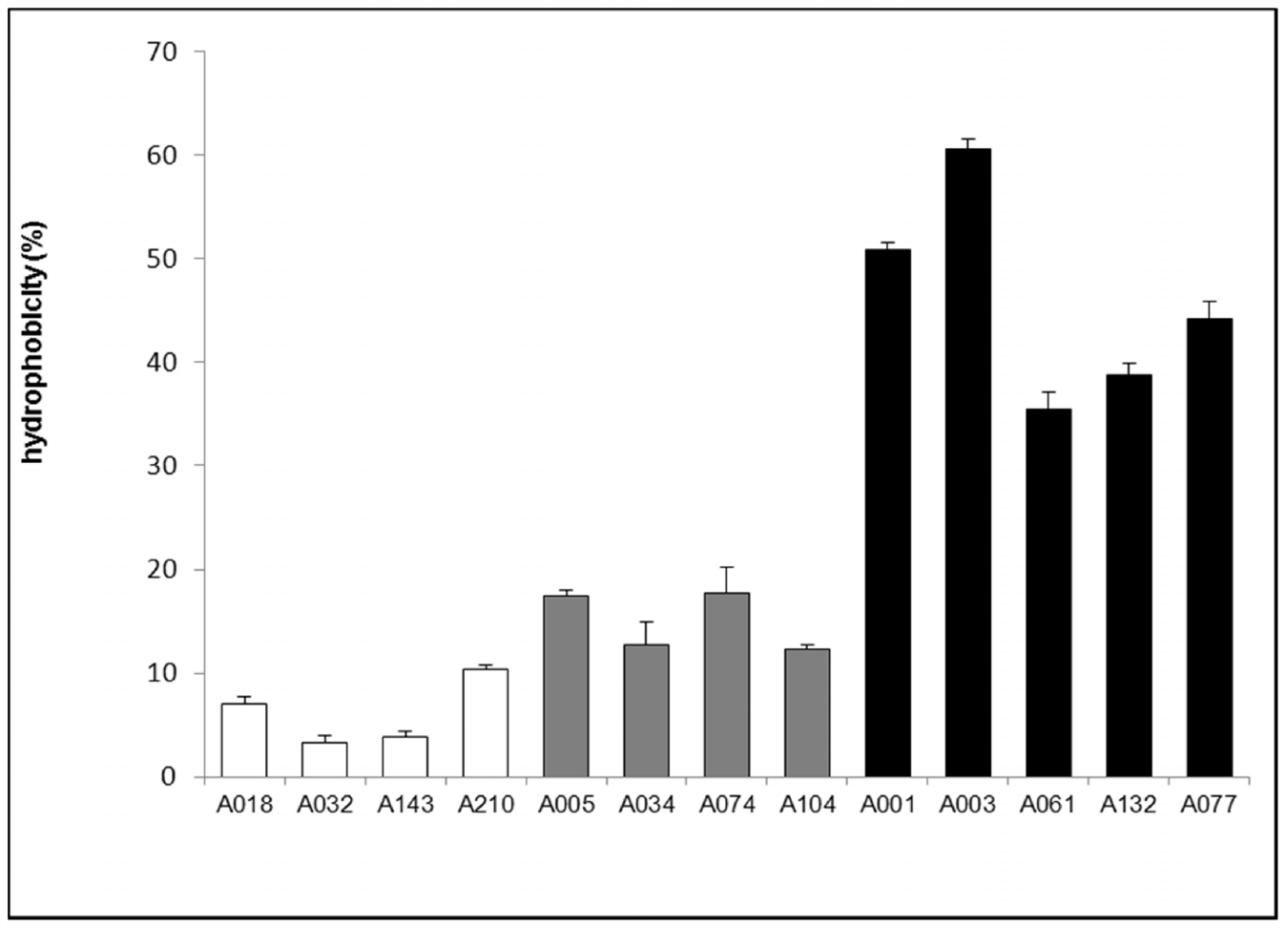
Bacterial hydrophobicity. Cellular hydrophobicity was measured and compared between non-biofilm forming (Pellicle – Biofilm -), pellicle-forming (Pellicle +) and solid biofilm-forming (Biofilm +) *A. baumannii* strains. Results are presented as means of at least 3 measurements for each strain. Error bars represent standard error with a 95% confidence interval. Results of ANOVA test, comparing hydrophobicity between the three groups, give p-values <0.001.

Cellular hydrophobicity was studied and compared between non-biofilm forming, pellicle-forming, and solid biofilm-forming (formed on the wall of the tube) *A. baumannii* strains by an extraction method in organic solvent (Figure 4). Pellicle-forming strains were markedly more hydrophobic (35–60% of the bacterial extraction by hexadecane) than solid biofilm-forming strains (12–17%) or non-biofilm forming counterparts (3–10%).

### Monosaccharide composition

**Figure 5:**
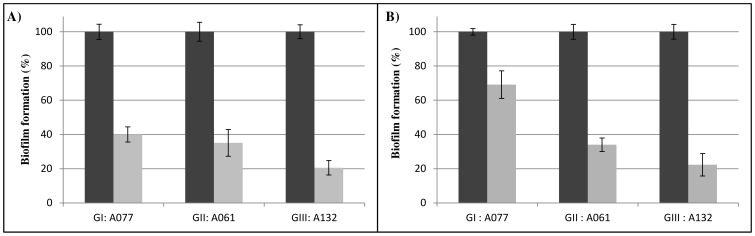
Effect of Dispersin B on pellicle formation for each morphotype. A077 strain from morphotype I, A061 strain from morphotype II and A132 strain from morphotype III in presence of 50 µg/mL Dispersin B (grey) or without Dispersin B (black). **A)** Dispersin B was added at the beginning of the pellicle growth and biofilm formation was quantified at 24 h by cristal violet assay; **B)** Dispersin B was added on a 24 h-pellicle and biofilm formation was quantified after additional 24 h. Results are presented as means (from at least 3 replicates for each condition). Error bars represent standard error with a 95% confidence interval. Results of ANOVA test, comparing the pellicle formation with and without Dispersin B for each strain, give p-values <0.001.

Gas chromatography analysis of the purified matrix polysaccharide was performed on 1 mg sample from each morphogroup representative strain (Table 1). Total carbohydrate analysis confirmed that pellicle matrices contained a carbohydrate-rich material; the main monosaccharide components were glucose and N-acetyl-glucosamine, with differences among groups (Table 1). Isolates from groups I and III showed a high conserved carbohydrate composition with a matrix composed mainly of glucose residues while the isolate A061 from group II contained above all N-acetyl glucosamine (Table 1). The activity of Dispersin B, which is an endoglycosidase specific to β-1-6-linked poly-glucosamine molecules [33] was significant on pellicle formation when it was added at the beginning of the culture (60 to 80% of decrease for all morphotypes, Figure 5A). The activity of the enzyme remained effective on 24-h pellicles (Figure 5B).

**Table 1:** Types and characteristics of pellicles produced by *A. baumannii*.

					Monosaccharide composition (% mol)					
Groups	Strain	Isolate source	MDR*	Hydrophobicity (%)	Glc	Kdo	GlcNAc	GalA	GalNAc	ManAc
**I – Egg-shaped**	A077	Urine	S	44±2	48.6±4.0	29.3±2.3	18.6±4.0	2.2±0.2	0.5±0.6	0.9±0.8
**II – Ball-shaped**	A061	Urine	MDR	35±2	28.1±2.5	11.0±1.3	51.5±3.6	–	9.4±1.0	–
**III – Irregular**	A132	Urine	S	38±1	43.3±5.0	27.6±1.9	25.0±4.9	1.9±0.3	1.5±1.3	0.6±0.5

( *^)^ S for sensitive, MDR for Multidrug-resistant as defined by Magiorakos *et al.* [52].

### Identification of matrix-associated proteins

**Figure 6:**
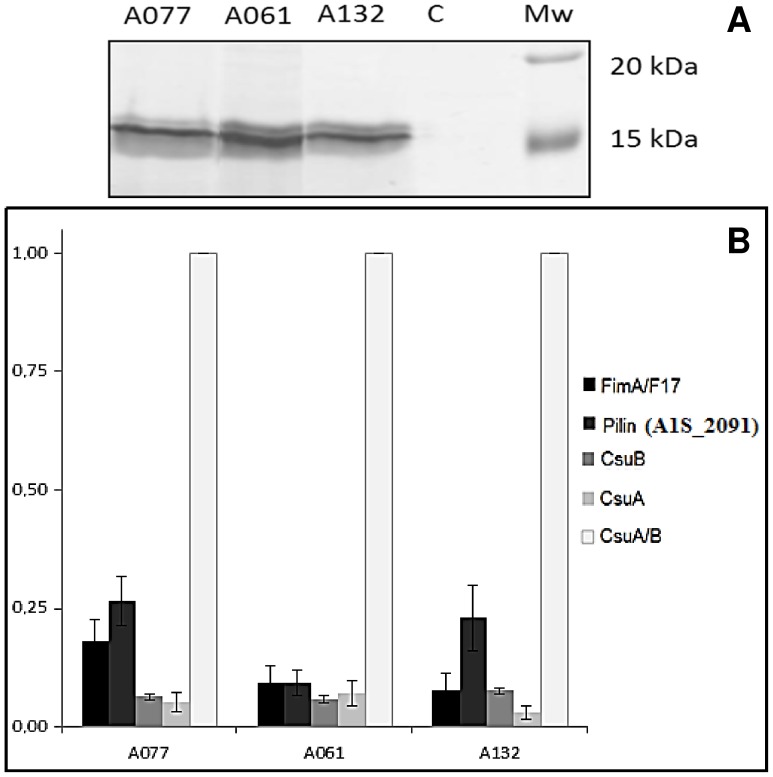
Matrix associated proteins of the pellicles formed by each representative strain of the three morphotypes. **A)** separated and visualized by SDS-PAGE and silver nitrate staining respectively, after treatment with 0.2% cellulase. **B)** Histogram giving relative abundances of proteins calculated according to the minimum-maximum normalization method from 3 biological replicates and 2 technical replicates. Error bars represent standard error with a 95% confidence interval.

Proteins embedded within the pellicle matrix were difficult to analyse due to the high amount of polysaccharides interfering with migration. Consequently, purified matrices were treated with cellulase previous to SDS-PAGE separation. No high molecular weight proteins were detected associated to the pellicle matrix but different low molecular weight proteins were identified in the samples from each morphogroup (Figure 6 & Table S2). Three proteic bands (located at about 15 kDa) were cut then digested and analysed by LC-MS/MS. The most abundant proteins were identified as pilins. As showed in the Figure 6 (and emphasized by psm values, see Table S2), CsuA/B (A1S_2218) was the most abundant protein in all matrices. This protein as well as CsuA (A1S_2217) and CsuB (A1S_2216), are secreted subunits of the CsuA/BABCDE chaperon-usher pili assembly system, which has been described to be involved in the attachment and biofilm formation of *A. baumannii* on abiotic surfaces [16]. Another identified pilin was the protein A1S_2091, which could be the major subunit of a chaperone–usher pili assembly (CU) system consisting in 4 proteins (A1S_2088-2091, reversed ORFs) in which A1S_2090 would be the assembly chaperone and A1S_2089 the fimbrial usher. After a sequence analysis and a BLASTP in the data bank, we found this CU system extremely well conserved in *A. baumannii* (>90% identity except in ACICU strain in which it was absent), and in the other *Acinetobacter* species, *i.e. A. pittii, A. nosocomialis* and *A. calcoaceticus* (>80% identity, see Table S3). Finally, the subunit coded by A1S_1507 gene was also identified as part of an operon coding for a last A1S_1510-1507 CU system, (again reversed ORFs in ATCC 17978). This CU system is constituted by the pilin FimA (A1S_1510 sometimes annotated F17-A protein in *A. baumannii* genomes), then a PapD chaperone (A1S_1509), a PapC porin (A1S_1508) and the tip adhesion pilin could be the A1S_1507 protein. This operon has already been shown to be involved in biofilm formation on solid support [34]. As shown by the Table S4, this operon is also well conserved in *A. baumannii* (except in AYE strain where the operon showed the insertion of an ISAbaI in A1S_1508 usher protein), and was found in ACB complex as well as in *A. junii*.

## Discussion

As for many bacterial species, the biofilm formation by *A. baumannii* has been linked to device-associated infections, antibiotic resistance and survival on dry surfaces [13], [35], [36]. Recent studies have also confirmed the capacity of this species to form pellicles at the air-liquid interface [20]–[22], reporting a higher frequency in *A. baumannii* and *A. nosocomialis* (formerly *Acinetobacter* genospecies 13TU), than in other less pathogenic *Acinetobacter* spp. [21].

Previous studies on *Pseudomonas* and *Salmonella* spp. described distinctive colony morphologies on congo red agar plates, identifying pellicle forming bacteria that produced dry and wrinkled colonies, defined as *rdar* in *Salmonella* spp. or wrinkly spreaders for *Pseudomonas fluorescens* [37]–[39]. This approach was not suitable with *A. baumannii* pellicle-forming isolates, since their growth, colour and morphology on congo red agar were highly variable. Consequently, pellicles were studied in liquid media to observe not only morphological differences among the strains, but also pellicle cohesion and matrix composition. The screening performed by optical microscopy on 26 pellicle-forming strains (30% of our collection), allowed their clustering into three morphological groups (Table 1, Figure 2). By contrast Koza *et al*. [5] classified *P. fluorescens* pellicles into four classes on basis of phenotype and physical robustness, considering their strength, sugar component and ability to attach at the meniscus. In our study, all the morphological groups were attached to a certain extent to the walls of the tube. Consequently, BAM microscopy examinations performed during the initial stages of pellicle formation aimed to elucidate whether this structure could develop without a solid anchor or bacteria attached to the wall were expanding and covering the liquid media as already described for *P. fluorescens* wrinkly spreaders [39]. Observations (Figure 3) pointed out that, contrary to *P. fluorescens* [39], *Acinetobacter* bacteria emerged anywhere on the liquid interface, formed aggregations that grew covering the whole surface and attached afterwards to the tube.

In wrinkly spreader *P. fluorescens*, bacterial recruitment to the air-liquid interface was associated to cell hydrophobicity combined with surface-interactions which would lead bacteria to be at the surface rather than submerged [5], [39]. Our results are consistent with these data; indeed, the bacterial pellicle forming ability was associated with high strain hydrophobicity (Figure 4). These results disagree with those of McQueary *et al.* who reported no correlation between cell hydrophobicity and pellicle formation in *A. baumannii* [22]. This discrepancy may be attributed to the temperature used in their assays (37°C), as pellicles in *A. baumannii* are more commonly observed at 25°C [20], [21]. This hydrophobicity that may be associated with an obligate aerobic status could explain why some *A. baumannii* strains were able to grow at the surface of the liquid media.

The microscopy analysis of the *A. baumannii* pellicles was followed by a chemical characterization of the matrix components. EPS matrix purification is a complex procedure since bacterial cells have to be separated from this carbohydrate rich substance and aggressive methodologies may damage bacterial cells leading to the release of cytoplasmic material [7]. In the present study, high molecular weight proteins were not detected and the identified matrix associated proteins were mostly pilin-like proteins. Although not directly considered as matrix components, these extracellular appendages are known to stabilize the structure, especially to maintain pellicles at the air-liquid interface, and have been widely related to the matrix [7], [8]. We confirmed that, at least, three pili systems are present in matrix pellicle and could be involved in the cohesion of the matrix structure. The first one is the Csu operon which is required for biofilm formation on solid surface [16] and would contribute to *A. baumannii* pellicle building, the CsuA/B pilin being the most abundant protein detected in matrix. The second pili system is a CU system from which we identified the pilin A1S_1510, annotated as FimA or F17aA protein, which presents 46% identity with the F17A pilin, member of F17A-G pili system expressed in *E. coli*. In enterotoxigenic *E. coli*, these pili mediate attachment to intestinal microvilli, leading to diarrhea or septicemia in ruminants [40], [41]. Disruption of the tip adhesin (A1S_1507) gene was shown to induce a severe decrease in biofilm formation on polypropylene plates [34]. Of note, the homologue of this operon in *A. baylyi*, the AcuADCG operon, was described to be involved in formation of thin pili required for adhesion to polystyrene and erythrocytes [42]. Finally, the identified pilin A1S_2091 is part of the 4-proteins CU system that has not been described before, which is extremely well conserved in the ACB complex but did not present any significant identity with some known systems.

Matrix exopolysaccharide composition has been shown to be complex (*e.g*., *P. aeruginosa* biofilms matrix containing among others Pel, Psl and alginate polysaccharides [43], [44]) but also variable in monomer units and linkages used to form the polysaccharide [8]. Cellulose-like polysaccharides, such as Pel in *P. aeruginosa* PA14 [30], rich in glucose subunits, are usually associated to solid biofilm structures and often to pellicle formation. Partially acetylated cellulose has also proved to be important for the formation of strong pellicles by wrinkly spreader *P. fluorescens* and *Salmonella* spp. [8], [25], [43]. In the present study, carbohydrate analysis corroborated the similarities of robustness observed between the morphogroups I and III, presenting a matrix composed mainly of glucose. In contrast, the isolate from the morphogroup II possess a matrix mainly composed of GlcNAc, an amino sugar with important structural roles in cell surface [45]. GlcNAc also forms part of a different type of surface polymer, PNAG, that plays an important role as virulence factor in staphylococcal biofilm formation [46] and has also been identified in numerous Gram-negative pathogenic bacteria. It has been recently reported in *A. baumannii* to be essential for maintaining the integrity of biofilm formed in dynamic environments [47]. In our case, the reactivity of Dispersin B (that specifically cleaves the β-(1,6)-linked N-acetylglucosamine polymer) on pellicle formation of each morphogroup suggests that their pellicle matrix may contain PNAG. Finally, the identification of a component as Kdo purified matrix revealed the presence of lipopolysaccharide (LPS), which instead of being a cellular contamination, could have been released into the external surrounding by the bacteria. In *P. fluorescens* pellicles, the interaction between LPS and cellulose gives strength and integrity, playing an important role in the relative hydrophobicity of the cell and bacterial attachment to surfaces [39].

In conclusion, as already observed in *P. aeruginosa* [48], the matrix of *A. baumannii* pellicles is complex, containing different extracellular polymeric substances, *i.e.* several exopolysaccharides, lipopolysaccharide, cell surface appendages (and probably eDNA, [49]), that may be contributing factors for the persistence and epidemicity of *A. baumannii* in the clinical environment [13], [35], [50]. In the hospital environment, pellicles could be formed in small droplets and therefore, might be involved in bacterial colonization of humidifiers, respiratory systems and any moist surfaces or apparatus already described as potential sources for *A. baumannii* isolation in hospital outbreaks [51]. Bacterial clusters may eventually be detached from the pellicle and move freely to cause infection if transfer into the patient occurs, *i.e.* through a respiratory system. As pellicles are more readily formed at 25°C than 37°C, bacteria in the pellicle could revert to the planktonic state and cause infection. For this reason, hospital antiseptics and disinfectants should target this biofilm structure, basically the matrix components that give stability and protect bacteria from external hazards.

## Supporting Information

Table S1 ***A. baumannii*** **clinical isolates used in this study.**
(DOCX)

Table S2 **Matrix associated proteins identified in the three morphotypes of pellicles produced by** ***A. baumannii.***
(DOCX)

Table S3 **Percentages of identity of A1S_2091–2088 proteins with homologues in related organisms.**
(DOCX)

Table S4 **Percentages of identity of A1S_1510–1507 proteins with homologues in related organisms.**
(DOCX)
