# Supplementary material for: Characterisation of Pellicles Formed by Acinetobacter baumannii at the Air-Liquid Interface
Source: PLoS One. 2014 Oct 31;9(10):e111660. doi: 10.1371/journal.pone.0111660 (PMC4216135; doi:10.1371/journal.pone.0111660)
Supplement: Table S1 — A. baumannii clinical isolates used in this study. (DOCX) [file pone.0111660.s001.docx]

**Table S1**. ***A. baumannii* clinical isolates used in this study.**

| Strain | Group | Origin | Sample | Infection | Reference |
| --- | --- | --- | --- | --- | --- |
| 77 | Eggs | Spain | Urine |  | 24 |
| 77778 | Channel | Spain |  |  | This paper |
| A132 | Channel | France | Urine |  | This paper |
| A15-43 | Eggs | Spain |  |  | 24 |
| Ac159 | Eggs | Spain | Exudate | Colonization | 23 |
| A212 | Balls | France |  |  | This paper |
| Ac001 | Eggs | Spain | Respiratory | Colonization | 23 |
| Ac002 | - | Spain | Respiratory | Colonization | 23 |
| Ac003 | Balls | Spain | Blood | Bacteremia | 23 |
| Ac005 | - | Spain | Respiratory | Pneumonia | 23 |
| Ac006 | - | Spain | Respiratory | Low respiratory tract | 23 |
| Ac008 | - | Spain | Respiratory | Pneumonia | 23 |
| Ac009 | - | Spain | Catheter | Colonization | 23 |
| Ac011 | - | Spain | Respiratory | Low respiratory tract | 23 |
| Ac012 | Balls | Spain | Exudate | Colonization | 23 |
| Ac014 | Balls | Spain |  |  | 23 |
| Ac016 | Channel | Spain | Urine | Colonization | 23 |
| Ac022 | - | Spain | Abscese | Infection | 23 |
| Ac023 | - | Spain | Exudate | Colonization | 23 |
| Ac024 | - | Spain | Respiratory | Infection | 23 |
| Ac025 | Balls | Spain | Respiratory | Colonization | 23 |
| Ac026 | - | Spain | Urine | Infection | 23 |
| Ac028 | Channel | Spain |  |  | 23 |
| Ac030 | - | Spain | Respiratory | Colonization | 23 |
| Ac031 | - | Spain | Exudate | Colonization | 23 |
| Ac032 | - | Spain | Urine | Colonization | 23 |
| Ac033 | Balls | Spain | Urine | Colonization | 23 |
| Ac034 | - | Spain | Urine | Colonization | 23 |
| Ac040 | - | Spain | Exudate | Colonization | 23 |
| Ac043 | - | Spain | Urine | Infection | 23 |
| Ac046 | - | Spain | Urine | Colonization | 23 |
| Ac048 | - | Spain |  |  | 23 |
| Ac049 | - | Spain | Urine | Colonization | 23 |
| Ac050 | Eggs | Spain | Urine | Colonization | 23 |
| Ac051 | - | Spain | Urine | Infection | 23 |
| Ac053 | Channel | Spain |  |  | 23 |
| Ac055 | Balls | Spain | Exudate |  | 23 |
| Ac059 | - | Spain | Exudate |  | 23 |
| Ac061 | Balls | Spain | Urine | Infection | 23 |
| Ac063 | - | Spain | Exudate | Infection | 23 |
| Ac066 | - | Spain | Exudate | Colonization | 23 |
| Ac068 | - | Spain | Blood | Infection | 23 |
| Ac070 | Balls | Spain | Blood | Infection | 23 |
| Ac071 | - | Spain | Catheter | Colonization | 23 |
| Ac072 | - | Spain |  | Colonization | 23 |
| Ac073 | - | Spain | Urine | Colonization | 23 |
| Ac074 | - | Spain | Respiratory | Low respiratory tract | 23 |
| Ac086 | Balls | Spain |  | Infection | 23 |
| Ac096 | Balls | Spain | Cephalo.Liquid | Infection | 23 |
| Ac104 | - | Spain | Respiratory | Colonization | 23 |
| Ac108 | - | Spain |  |  | 23 |
| Ac110 | Channel | Spain |  |  | 23 |
| Ac112 | - | Spain | Respiratory | Pneumonia | 23 |
| Ac116 | - | Spain | Respiratory | Pneumonia | 23 |
| Ac117 | - | Spain | Respiratory | Colonization | 23 |
| Ac135 | - | Spain | Respiratory | Colonization | 23 |
| Ac137 | - | Spain | Respiratory | Colonization | 23 |
| Ac141 | - | Spain | Catheter | Colonization | 23 |
| Ac142 | - | Spain | Respiratory | Low respiratory tract | 23 |
| Ac149 | - | Spain | Respiratory | Pneumonia | 23 |
| Ac154 | - | Spain | Urine | Infection | 23 |
| Ac156 | - | Spain | Cephalo.Liquid | Meningitis | 23 |
| Ac163 | - | Spain | Respiratory | Colonization | 23 |
| Ac160 | - | Spain | Respiratory | Pneumonia | 23 |
| Ac162 | - | Spain | Urine | Colonization | 23 |
| Ac164 | - | Spain | Blood | Bacteremia | 23 |
| Ac166 | - | Spain | Urine | Colonization | 23 |
| Ac169 | - | Spain | Exudate | Infection | 23 |
| Ac172 | Eggs | Spain | Urine | Infection | 23 |
| Ac175 | Balls | Spain |  |  | 23 |
| Ac178 | Balls | Spain | Exudate | Infection | 23 |
| Ac183 | - | Spain | Respiratory | Colonization | 23 |
| Ac186 | - | Spain | Exudate | Infection | 23 |
| Ac192 | - | Spain | Skin | Infection | 23 |
| Ac198 | Eggs | Spain |  |  | 23 |
| Ac199 | - | Spain | Urine | Colonization | 23 |
| Ac207 | - | Spain | Urine | Colonization | 23 |
| Ac208 | - | Spain | Urine | Colonization | 23 |
| Ac210 | - | Spain | Exudate | Colonization | 23 |
| Ac213 | - | Spain | Blood | Colonization | 23 |
| Ac215 | - | Spain | Respiratory | Colonization | 23 |
| Ac223 | - | Spain | Exudate | Colonization | 23 |
| Ac233 | - | Spain | Respiratory | Colonization | 23 |
| Ac237 | - | Spain |  |  | 23 |
| Ac244 | - | Spain | Respiratory | Colonization | 23 |
| Ac245 | - | Spain | Blood | Infection | 23 |
