# Supplementary material for: Characterisation of Pellicles Formed by Acinetobacter baumannii at the Air-Liquid Interface
Source: PLoS One. 2014 Oct 31;9(10):e111660. doi: 10.1371/journal.pone.0111660 (PMC4216135; doi:10.1371/journal.pone.0111660)
Supplement: Table S2 — Matrix associated proteins identified in the three morphotypes of pellicles produced by A. baumannii. (DOCX) [file pone.0111660.s002.docx]

| **Locus_ tag** | **Description** | **PSM* A077** | **PSM A061** | **PSM A132** | **Mw (kDa)** |
| --- | --- | --- | --- | --- | --- |
| **A1S_2218** | **putative protein CsuA/B; secreted protein type I pili** | **406** | **399** | **429** | **18.7** |
| **A1S_2091** | **conserved hypothetical protein** | **37** | **101** | **90** | **15.0** |
| **A1S_1510** | **fragment of putative fimbrial protein precursor (Pilin)** | **35** | **67** | **28** | **12.6** |
| A1S_0779 | conserved hypothetical protein | 30 | 39 | 68 | 18.9 |
| A1S_1183 | conserved hypothetical protein | 28 | 47 | 39 | 15.2 |
| **A1S_2217** | **protein CsuA** | **27** | **19** | **11** | **20.8** |
| A1S_3384 | conserved hypothetical protein | 23 | 52 | 45 | 17.5 |
| A1S_2213 | putative protein CsuE; secreted protein related to type I pili | 23 | 26 | 34 | 37.3 |
| A1S_1966 | FabZ (3R)-hydroxymyristoyl-[acyl carrier protein] dehydratase | 23 | 16 | 0 | 18.0 |
| **A1S_2216** | **putative protein CsuB; secreted protein type I pili** | **22** | **25** | **32** | **16.0** |
| A1S_3075 | RpsC 30S ribosomal protein S3 | 16 | 64 | 5 | 27.9 |
| A1S_2773 | putative long-chain fatty acid transport protein | 15 | 26 | 19 | 53.1 |
| A1S_0597 | RplT 50S ribosomal protein L20. | 12 | 54 | 17 | 13.4 |
| A1S_0869 | TufA protein chain elongation factor EF-Tu | 11 | 45 | 0 | 41.2 |
| A1S_0820 | conserved hypothetical protein | 10 | 47 | 19 | 16.9 |
| A1S_2711 | SdhC succinate dehydrogenase. cytochrome b556 subunit | 10 | 29 | 14 | 14.5 |
| A1S_2167 | CyoB cytochrome o ubiquinol oxidase subunit I | 10 | 11 | 0 | 74.5 |
| A1S_0835 | LolB outer-membrane lipoprotein precursor | 9 | 7 | 9 | 21.0 |
| A1S_3343 | conserved hypothetical protein; putative exported protein | 8 | 8 | 0 | 17.3 |
| A1S_0867 | RpsG 30S ribosomal protein S7 | 7 | 50 | 2 | 17.7 |
| A1S_0292 | putative outer membrane protein W | 4 | 11 | 0 | 22.6 |
| A1S_0285 | RplJ 50S ribosomal protein L10 | 0 | 54 | 0 | 18.1 |
| A1S_3062 | RplO 50S ribosomal protein L15 | 0 | 46 | 0 | 15.5 |
| A1S_3001 | RpsI 30S ribosomal protein S9 | 0 | 39 | 0 | 14.3 |
| A1S_3057 | RpsD 30S ribosomal protein S4 | 0 | 36 | 0 | 23.3 |
| A1S_2710 | GltA citrate synthase | 0 | 33 | 0 | 47.4 |
| A1S_3069 | RplE 50S ribosomal protein L5 | 0 | 33 | 0 | 20.0 |
| A1S_0151 | AtpF membrane-bound ATP synthase. F0 sector. subunit b | 0 | 32 | 0 | 17.0 |
| A1S_2834 | mscL mechanosensitive channel | 0 | 26 | 5 | 15.8 |
| A1S_1338 | hypothetical protein | 0 | 0 | 22 | 25.4 |
| A1S_3074 | RplP 50S ribosomal protein L16 | 0 | 18 | 0 | 15.5 |
| A1S_2809 | EcnB bacteriolytic lipoprotein entericidin B. | 0 | 0 | 16 | 5.0 |
| ***PSM: peptide spectral matches** | |  | | |  |

**Table S2. Matrix associated proteins identified in the three morphotypes of pellicles produced by *A. baumannii***
