# Supplementary material for: Characterisation of Pellicles Formed by Acinetobacter baumannii at the Air-Liquid Interface
Source: PLoS One. 2014 Oct 31;9(10):e111660. doi: 10.1371/journal.pone.0111660 (PMC4216135; doi:10.1371/journal.pone.0111660)
Supplement: Table S3 — Percentages of identity of A1S_2091–2088 proteins with homologues in related organisms. (DOCX) [file pone.0111660.s003.docx]

**Table S3**. **Percentages of identity** **of A1S_2091-2088 proteins with homologues in related organisms**.

| ***A. baumannii* ATCC17978 operon** | **A1S_2091**  **Pilin**  **17.95 kDa** | **A1S_2090**  **assembly chaperone**  **26.17 kDa** | **A1S_2089**  **fimbrial usher**  **91.01 kDa** | **A1S_2088**  **Adhesin**  **35.98 kDa** |
| --- | --- | --- | --- | --- |
| ***A. baumannii* ATCC19606** | **100%***  ACIB1v1_70066 | **99.15%** ACIB1v1_70067 | **99.5 %** ACIB1v1_70068 | **99.7%** ACIB1v1_70069 |
| ***A. baumannii* AYE** | **100%**  ABAYE1470 | **99.15%**  ABAYE1471 | **99.6 %**  ABAYE1472 | **100%**  ABAYE1473 |
| ***A. nosocomialis* NIPH 2119** | **88%**  N8SQU2_9GAMM | **87%**  N8RBK8_9GAMM | **85%**  N8RBK2_9GAMM | **80%**  N8SPJ9_9GAMM |
| ***A. pittii* CIP 70.29** | **91%**  N9G1G1_ACIG3 | **84%**  N9EKY6_ACIG3 | **87%** N9EKC0_ACIG3 | **91 %**  N9G1G3_ACIG3 |
| ***A. calcoaceticus* RUH2202** | **84.7%** ACICAv1_280217 | **85 %** ACICAv1_280216 | **87.55 %** ACICAv1_280215 | **92.8 %**  ACICAv1_280214 |
|  | **81.8%** ACICAv1_280156 | **74 %** ACICAv1_280155 | **84.35 %** ACICAv1_280154 | **74.55 %** ACICAv1_280153 |
| ***A. baylyi* ADP1** | **29.9 %**  ACIAD3336 | **31.5%**  ACIAD3335 | **24.8%**  ACIAD3334 | **30.1 %**  ACIAD3333 |
| ***A. lwoffii* SH145** | − | − | − | − |
| ***A. junii* SH205** | _ | − | − | − |
| ***A. johnsonii SH046*** | − | − | − | − |
| ***P. aeruginosa* PAO1** | **30.7 %**  PA4650 | **26.7 %**  PA4651 | **29.5 %**  PA4652 | **27.55 %**  PA4653 |
|  | **24.7 %**  PA0992 (CupC1) | **23.2 %**  PA0993 (CupC2) | **24.2 %**  PA0994 (CupC3) | − |
| ***E. coli* 536** | **25.0%**  ECP_4534 | − | − | − |

(*) Percentages are obtained with BlastP software at Genoscope and Uniprot databases.
