# Supplementary material for: Characterisation of Pellicles Formed by Acinetobacter baumannii at the Air-Liquid Interface
Source: PLoS One. 2014 Oct 31;9(10):e111660. doi: 10.1371/journal.pone.0111660 (PMC4216135; doi:10.1371/journal.pone.0111660)
Supplement: Table S4 — Percentages of identity of A1S_1510–1507 proteins with homologues in related organisms. (DOCX) [file pone.0111660.s004.docx]

**Table S4**. **Percentages of identity** **of A1S_1510-1507 proteins with homologues in related organisms**.

| ***A. baumannii* ATCC17978**  **operon** | **A1S_1510**  **Pilin FimA/F17a**  **18.36 kDa** | **A1S_1509**  **Chaperone PapD**  **27.97 kDa** | **A1S_1508**  **Porin PapC**  **96.03 kDa** | **A1S_1507**  **Adhesin**  **36.40 kDa** |
| --- | --- | --- | --- | --- |
| ***A. baumannii* ATCC19606** | **100%** ACIB1v1_200027 | **98.8%** ACIB1v1_200028 | **98.9%** ACIB1v1_200029 | **99.4%** ACIB1v1_200030 |
| ***A. baumannii* ACICU** | **99.2%**  ACICU_01551 | **100%**  ACICU_01550 | **98.4%**  ACICU_01549 | **99.4%** ACICU_01548 |
| ***A. nosocomialis* NIPH 2119** | **95%** N8RAC7_9GAMM | **89%** N8RAD1_9GAMM | **90%** N8SPM2_9GAMM | **87%** N8SJS8_9GAMM |
| ***A. pittii* CIP 70.29** | **89%** N9F335_ACIG3 | **74%** N9GIA4_ACIG3 | **70%** N9GBP2_ACIG3 | **82%** N9GIF9_ACIG3 |
| ***A. calcoaceticus* RUH2202** | **90.1 %** ACICAv1_210032 | **76.1 %** ACICAv1_210031 | **73.4%** ACICAv1_210030 | **84.6 %** ACICAv1_210029 |
| ***A. baylyi* ADP1** | **29.4%** ACIAD0387 | **37.8 %**  ACIAD0388 | **27.8 %** ACIAD0389 | **28.3 %** ACIAD0390 |
| ***A. lwoffii* SH145** | − | − | − | − |
| ***A. junii* SH205** | **76.55%** ACIJUv1_520035 | **65.3%** ACIJUv1_520036 | **62.6%** ACIJUv1_520037 | **55.95%** ACIJUv1_520038 |
| ***A. johnsonii* SH046** | − | − | − | − |
| ***P. aeruginosa* PAO1** | **29.20%**  PA0992 | **37.2%**  PA4085 | **35.65%**  PA4084 | **21.16%**  PA5284 |
| ***E. coli* 536** | **44.5 %**  ECP_3785 | **42.15 %**  ECP_3784 | **33.75**  ECP_3783 | **23.27 %**  ECP_3782 |

(*) Percentages are obtained with BlastP software at Genoscope and Uniprot databases.
